# Widening the scope of mental health with a ‘youth centred’ approach: a qualitative study involving health care professionals in Sweden’s youth clinics

**DOI:** 10.1080/17482631.2024.2348879

**Published:** 2024-05-03

**Authors:** Isabel Goicolea, Linda Richter Sundberg, Maria Wiklund, Anne Gotfredsen, Monica Christianson

**Affiliations:** a Department of Epidemiology and Global Health, Umeå International School of Public Health, Umeå University, Umeå, Sweden; b Department of Psychology, Umeå University, Umeå, Sweden; c Department of Community medicine and rehabilitation, Physiotherapy, Umeå University, Umeå, Sweden; d Department of Nursing, Umeå University, Umeå, Sweden

**Keywords:** Youth mental health, youth-centredness, qualitative, reflexive thematic analysis, interviews, youth clinic

## Abstract

**Purpose:**

The aim of this study was to explore how health care providers at youth clinics (YCs) in Sweden engage with, focus on, and navigate across the mental health youth space, while upholding the core bedrock principle of “youth-centeredness”.

**Methods:**

Qualitative interviews were conducted with 21 health care professionals working in three YCs located in three different regions of Sweden. Data were analysed using reflexive thematic analysis informed by the work of Braun and Clarke.

**Results:**

The three themes were: 1) “youth mission—at the core of the YCs” work and challenged by a stronger involvement in mental ill health’; 2) “YCs” unique and complementary role in the youth mental health system: a holistic perspective, team work, and a focus on normalization’, and 3) “Caught between a rock and a hard place: to treat at a care level that is not optimal for the young users” needs or to refer within an unreliable system’.

**Conclusion:**

This study reflects the individuality and key features of YCs, their widening roles within the mental health sphere, and the challenges faced in maintaining and expanding the characteristic “youth-centred” approach while expanding their work with mental health.

## Introduction

Youth clinics (YCs) in Sweden have been operating with a youth-centred approach since the 1970s. By youth-centred we mean that “YCs” address the diverse needs that each young user may have by means of a holistic approach to health, which includes aspects related to mental health and wellbeing’ (2, p 2). Young people have free access to these clinics, where consultations are needs-based and waiting times are kept to a minimum (Föreningen för Sveriges Ungdomsmottagningar, 2015, 2018b; Thomée et al., 2016). Information and consultations concerning mental health have been included in YCs’ work from the very beginning (Goicolea et al., 2018; UMO, 2023), and since the 2010’s some YCs are included in the first-line mental health care for young people. This means that they are part of the health care services that first encounter young people with signs of mental health problems, in order to make an initial assessment and provide appropriate care and support (Föreningen för Sveriges Ungdomsmottagningar, 2018a; Sveriges Kommuner och Regioner, 2022). This qualitative study investigates how health care professionals in Sweden’s YCs work in the mental health space and draws lessons from these experiences.

### Sweden’s youth clinics: an important arena for youth mental health care

Sweden’s YCs are more prevalent and have greater coverage compared with similar services in other high-income countries (e.g., Australia’s “HeadSpace”, Ireland’s “Jigsaw Clinics”) (Hetrick et al., 2017; McGorry, 2019; McGorry et al., 2013; O’keeffe et al., 2015; Rickwood et al., 2019). Sweden has approximately 257 YCs (UMO, 2023) which meet the health and other care needs of young people—aged 12 to 25 years, although age limits differ in different clinics. A core objective is to promote physical and mental health and well-being, with a focus on sexual and reproductive health and rights (Föreningen för Sveriges Ungdomsmottagningar, 2018a, 2018b). Midwives, social counsellors, psychologists and physicians are core professionals; nutritionists, nurses and physiotherapists are included in some YCs’ teams. Consultations do not have user fees; clinics are typically open Monday through Friday and are often placed in the city centre (Föreningen för Sveriges Ungdomsmottagningar, 2018a).

Our previous research highlights how Sweden’s YCs’ policies and practices are consistent with the World Health Organization (WHO) five criteria of youth-friendliness—i.e., accessibility, acceptability, equity, appropriateness and effectiveness (Sundberg et al., 2021; Thomée et al., 2016; Uppdrag Psykisk Hälsa, 2017). This is remarkable, since worldwide most services targeting young people struggle to achieve this (Tylee et al., 2007). Yet there have been calls for improvements in YCs, for example in regard to (in)equalities and (in)equities in access (Radez et al., 2021; Thomson et al., 2022; Waenerlund et al., 2020).

Since the early 2010s, a small proportion of Sweden’s YCs have had a formal responsibility to provide first-line mental health care (Föreningen för Sveriges Ungdomsmottagningar, 2018b). In 2016 YCs were allocated extra resources, with the aim of promoting mental health and preventing mental illness among young people. This came with changed responsibilities and extra workloads in processes, routines and documentation (Baroudi et al., 2020; Mosquera et al., 2017). Yet there has been a lack of clarity around mental health responsibilities, and practices often differ between the clinics (Goicolea et al., 2022; Socialstyrelsen, 2018). In some YCs, the emphasis is on the promotion of mental health and the prevention of ill health, while others focus more on the support and treatment of mental illness (Goicolea et al., 2018, 2022).

Although youth mental health care is a policy priority in Sweden, there are recognized weaknesses in existing mental health care services both in Sweden and elsewhere. It is therefore important to develop lessons from the way in which established models function in order to better understand the challenges and opportunities they face. The aim of this study was to explore how health care providers at YCs in Sweden engage with, focus on, and navigate across the mental health youth space, while upholding the core bedrock principle of youth-centredness.

## Methodology

This study is part of a larger study analysing the role of youth clinics on mental health from the perspective of professionals working in YCs, but also collaborating actors and young people. The larger study focused on three Swedish regions as cases. For this specific study we conducted and analysed interviews with 21 professionals with experiences working in 15 different YCs, representing all three regions. We used reflexive thematic analysis to analyse the transcripts (Braun & Clarke, 2006, 2022). The analysis was mainly inductive, but we were inspired by the WHO framework on youth friendliness (Uppdrag Psykisk Hälsa, 2017), as well as by conceptual debates around mental health; for example, frictions between biomedical conceptualizations on mental health and illness versus holistic approaches to mental health (Baroudi et al., 2020; Bianchi, 2016; Clark, 2014; Keyes, 2005; Rayner et al., 2018; Thompson, 2018).

### Participants and data collection

Participating YCs were located in the southern, central, and northern regions of Sweden. The YCs differed in relation to size and staffing as well as formal first-line assignment for youth mental health (only region 3 had this assignment).

We first contacted the YCs manager in the region to inform about the study and planned for a first meeting with professionals working in the YCs. During that meeting IG, LRS and AG presented the study and advised those interested in participating in the study to contact the research team via email to plan an interview. They were given written information covering the purpose of the study, rights and protections. All those who were invited freely gave their written informed consent.

Data collection was carried out sequentially in the different regions. We started with region 1, then moved to region 2 and finally region 3.

Since we wanted to get the perspective not only of the professionals who bear the main responsibility for mental health (namely counsellors and psychologists), we invited respondents from different professions to participate. This was done in order to understand how different professions work with mental health (e.g., the role of counsellors or psychologists can differ from that of midwives or doctors).

Twenty-one participants were interviewed across the three YCs. Table I gives a description of the participants from each clinic/region. While the basis for recruitment were three main YCs, the professionals interviewed worked in other clinics as well—for example in region 1 and 3 professionals rotated between the different YCs existing in the region, and in region 2 professionals rotated among two different clinics. That means that participants brought to the interviews their experiences from working in a total of 15 clinics.

**Table I.** Overview of participants from YCs (*n* = 21).

Region 1	Region 2	Region 3
**7 participants**	**9 participants**	**5 participants**
2 Midwives 1 Physician 3 Social counsellors 1 Director of all YCs in the region	2 Psychologists 1 Nurse/Sexologist 3 Social counsellors 1 Nurse 1 Branch head for two YCs in the region 1 Director for all YCs in the region	2 Social counsellors 1 Social counsellor and branch head for the psycho-social care 1 Midwife and branch head for the medical health care 1 Director for all YCs in the region

Five researchers experienced in qualitative research (IG, LRS, MW, MC, AG) conducted the interviews in Swedish. Three interviews were face-to face and eighteen were conducted digitally using Zoom software. The interviews lasted approximately one hour and were transcribed verbatim using a professional transcription service.

### Data analysis

The analysis was conducted in accordance with reflexive thematic analysis as described by Braun and Clarke (Braun & Clarke, 2006, 2022). We started by in-depth repeated reading of the transcripts before coding the material into descriptive candidate themes. After we developed our candidate themes, we wrote descriptions of these, and paired them with excerpts from the text that exemplified each candidate theme. We then went back to the transcripts to revise whether the candidate themes reflected and comprised the material. This process included repeated meetings within the research team (IG, MC, MW, LRS, AG) to discuss the analysis and preliminary writings. In addition to the meetings in the research team, we also held three meetings (one per YC) to discuss our preliminary results. Ongoing discussions evolved until final agreement was reached regarding the structure and assignment of the themes and their labels. In sum, we followed Braun and Clarke’s six steps, with the only difference, that writing was conducted hand in hand as part of the analysis, and not as a final step.

It is also important to mention, that the analysis was conducted in parallel to data collection and sequentially. This meant that after conducting the interviews in region 1, we did a preliminary analysis that further guided data collection in region 2 and afterwards region 3. This sequential process where data collection and analysis took place simultaneously allowed us to stop collecting data when we considered that: 1) the findings allowed us to answer the research questions, and 2) there started to appear many repetitions in the answers of the participants during the interviews.

The project received approval from the Ethics Review Authority in Sweden (Dnr 2019–2910, 2020–04720).

## Results

The results are grouped under three themes: 1) youth mission—at the core of the YCs’ work and challenged by a stronger involvement in mental ill health; 2) YCs’ unique and complementary role in the youth mental health system: a holistic perspective, team work, and a focus on normalization, and 3) Caught between a rock and a hard place: to treat at a care level that is not optimal for the young users’ needs or to refer within an unreliable system?.

### Youth mission—at the core of the YCs’ work and challenged by a stronger involvement in mental ill health

The so-called “youth mission” is central to the work of YCs. This refers to the way in which they encourage young people to define the reason for their presentation from the outset. This allows the consultation to focus on what the young person feels is important and wants to discuss.

I think we focus on the “youth mission”; what does the young person bring [to the consultation]? Validate that. Focus on what the youth also have as strengths. They get to participate in that process. That we do not decide over their heads. It is a dialogue; we try to have an equal balance of power in the contact. And then, I think they feel heard. (Social counsellor, Region 2)

As expressed in the above quote, the “youth mission” is about focusing on “strengths” rather than on problems. While this represents young people in a positive light, moving away from deficitary discourses on youth, it can also imply that problems are associated with weaknesses. The “youth mission” means that the young person’s complex needs are central.

We set up a treatment together. Then I can make suggestions, but what will be most important to start with is what the young person wants. And that’s the main difference with [other] health care [services], where they focus on diagnosis, and where they offer maybe CBT [cognitive behavioural therapy] or something to address the symptoms […] Here [at the YC] they can come and say “I have anxiety and I want to talk about the relationship with my boyfriend” or “my girlfriend”, and then that’s what we talk about. (Psychologist, YC2)

As the previous quote exemplifies, acknowledging the young person as an active agent means that they can set the focus and decide what they want to share. Hence the young person is at the centre of the consultation and the professional adapts accordingly.

Participants added another layer to this perspective. Being an active agent also means that the young person must take responsibility for their own health and well-being. The professional and the young person need to collaborate in finding a solution, but the young person needs to take a proactive role, e.g., making changes in habits such as diet, sleep, etc.

However, the interviews also identified flexibility within YCs in relation to this proactive role expected from youths. There were instances cited when professionals considered that they needed to take a more leading role because young people were feeling very low. This included relieving the young person of total responsibility for actively seeking and taking care of their own personal good health. Staff at YCs engaged in balancing acts between, on the one hand, respecting the autonomy of young people and approaching them as active agents and on the other hand, showing concern and being somewhat “motherly” at times.

Another important aspect of the “youth mission” was that YCs should keep a “low threshold” for access, meaning that nothing is too small or too big to discuss.

So, in everything, we try to say that no questions are too big or too small. “Come to us, we’ll try to solve it”. And, of course, we can’t solve everything here, but then we can at least help… If you don’t know where to go, you can always come to the YC and we’ll try to help. (Manager, YC3)

In Sweden, YCs cover medical, social, psychological, reproductive and sexual issues. Given the low access threshold, young people aged 12 to 25 years, present with a diversity of mental health problems and illnesses to YCs. For the staff this is challenging; clients with more severe conditions require longer hours of contact and follow-up which adds to waiting times for others. They acknowledged the trade-off between maintaining open access and keeping wait times low.

Juggling the YC “youth mission” with increasing responsibilities for mental health was seen in regard to conflicting discourses concerning the prioritization of youth over parents.

We work on behalf of the young people. So, if you have more difficult concerns and need a psychological examination or a diagnosis […] or something where you need parents with you, then we are not the right place. Because we can’t work with the family like that; our mission is to work on behalf of the young people. (Physician, YC1)

Like the physician’s quoted above, participants also reflected on the fact that the treatment of mental illnesses may require stronger involvement by parents, especially when a young person is under the age of eighteen. This brings a challenge to YCs that have always been cautious regarding parental involvement; in YCs parents have traditionally played a secondary role to allow the focus to be on young people. It is both that a core value of YCs is that it is the young person who is in focus and an active agent, but also that in practical terms, the presence of parents and other adults in the waiting room may be perceived as intrusive by some young people waiting for consultations. The YC principle differs from that at child and adolescent psychiatry services, where parental involvement is expected.

The “youth mission” speaks to the principal that YCs are safe places for young people to build trusting relationships with professionals.

But precisely by giving space for what is important in their lives, and to strengthen their agency, strengthen their own will and desires, in a world that often is controlled and regulated by adults […]. So, I still see that as a big part of my role here. (Psychologist, YC2)

These safe places are seen as being crucial for offering good accessible care, and maintaining a high level of satisfaction with the service. There was an understanding that a young person who seeks help at a YC (and also at other services) will be more likely to seek care throughout their lives if early experiences in regard to mental health consultations are positive.

The young person’s good experience at the YC is vital, since it provides opportunities for them to find a safe place in an adult-centred world. Experiencing safety, being treated with respect, and getting help on their own terms were essential beneficial contributors to mental well-being.

### YCs’ unique and complementary role in the youth mental health system: a holistic perspective, teamwork, and a focus on normalization

The way YCs work with mental health is different from that of other health and support services offered for young people.

Then it was a girl who called, […] and she wanted contraceptives. […] And then this girl says that “I feel much better now than I did in September”, and then the midwife asked; “what happened in September?” And she starts talking: “[…] I have a very difficult time […]” And she had made a suicide attempt in September […] Then I checked with my colleagues at the YC, and a social worker had a cancellation, so she had her first visit the same day and was extremely pleased. […] This is classic, what we are good at. In a contraceptive visit, you pick up something else. (Manager, YC1)

In the previous quote, the midwife discovered underlying mental health issues from what began as a request for contraceptives. It is a good example of how professionals at the YC not only address the reason for young peoples’ visits, but they also adopt a holistic approach and explore what may be behind the overt reasons for the visit. This includes making an assessment as to whether there is something else which may not be obvious.

This skill of digging deeper or going beyond the reason for consultation in a relaxed and casual way is at the very core of YCs. Participants described how YCs were spaces where young people feel comfortable to share experiences that otherwise may not be shared. They also commented that they, as professionals, observe experiences and situations that the young person may not see as being important or necessary to address.

To ensure such a holistic approach having a multidisciplinary team with a shared vision beyond the specific expertise of each professional group, was seen as being crucial.

But that many times the first contact with the YC may be the midwife because you want birth control pills, or you have questions about sex, or you want to pick up condoms or something like that. And then it’s an entrance to also… “yes, but how do you feel in general?” What about your mental health?” […] So, our role as midwives becomes very important to open up that possibility and to be able to refer further to our colleagues who work with counselling. (Midwife, YC2)

As exemplified in the previous quote, it was not only psychosocial staff dealing with issues related to mental health; all staff were a part of this work. The fact that the professionals together explored different aspects of the same problem was important in bringing a holistic approach for better understanding the concerns and problems being presented. This teamwork also meant that staff who did not focus solely on mental health felt supported; there were counsellors and psychologists to consult when needed. A major benefit was that individual professionals could learn from one other and develop their own skills.

The holistic perspective also meant that mental health is not conceptualized as a separate entity distinct from other aspects of health. The connection between mental health and sexual health was highlighted in the interviews. Participants emphasized that having the focus on sexual and reproductive health created opportunities for the holistic approach. They saw sexual and reproductive health consultations as a gateway to the YC. Presenting to the YC opens opportunities for the young person to discuss other issues.

A holistic approach also means focusing on “normalizing” young people’s mental health problems while at the same time confirming their feelings.

There are many people who come saying… “I have a panic disorder” […] And sometimes it’s not about panic disorder, it’s about strong emotional expressions and that we also try to understand what it’s about. It’s no wonder that you feel so strongly about this with everything going on in your life, and when there hasn’t been anyone who has been able to help you with it either. (Psychologist, YC2)

Participants also described how young people presenting at YCs refer to their symptoms in the form of psychiatric diagnoses. They explained how it is important to validate symptoms as being normal but not trivial or negligible. Staff tried to avoid using psychiatric labels for various conditions but instead saw needs and symptoms as a normal part of a young person’s development.

Professionals working in YCs with first line assignment for mental health encountered and treated a much wider spectrum of mental illnesses and psychiatric conditions, than others without first line assignment. This sometimes required a different approach.

Now I’m into this very thing with different levels of care. But like, not everything is normal. Some things are actually morbid […]. It’s not normal to try to kill yourself (Manager, YC3)

As the manager above describes, not everything can be normalized. The quote also revealed how the staff were perceived to have fundamental skills in separating young people’s normal reactions and emotions in response to life events from conditions that require referrals to specialist care.

### Caught between a rock and a hard place: to treat at a care level that is not optimal for the young users’ needs or to refer within an unreliable system

Participants stressed that YCs could not take responsibility for addressing the full spectrum of mental ill health possibilities. The range of mental health problems that YCs could manage within their resources varied.

The YCs with first line assignment had more resources and a formal obligation to take on mild to moderate levels of mental illness. The first line assignment also meant that professionals could keep and treat more young people at the YC because they could offer diagnosis and treatment so that fewer young people had to be referred to another service elsewhere.

Even if having the first line assignment (and accompanying resources) widened the scope of mental health problems, it was not possible to address all mental health needs. Collaboration with other services and having a well-functioning referral system was therefore necessary. However, setting and keeping limits in relation to who to treat and who to refer was not always easy.

Yes, but for example if we talk to someone at BUP [Child and adolescent psychiatry] and there is a person here who has the same education as that person at BUP, they might not really understand, “Yes, but why are you sending this to us? You have the same competence, why can’t you solve this problem?” […] We know that today there are very long waiting times in many other services. Then it is easy to sometimes slip over that limit. [and keep the young person at the YC]. (Social counsellor, YC1)

It was frustrating for professionals with the skills for diagnosis and treatment to be constrained in applying their skills. Psychosocial staff seldom knew very much about a young person at the initial meeting. If the discussion led to serious problems such as suicidal thoughts and self-harm, a referral to other services could be perceived as abandonment by the young person. Yet when there was mistrust in the capacity of specialized services to properly and promptly meet the young person’s urgent needs, the decision to either refer or keep the young person at the YC, also posed an ethical dilemma and created stress. Finally, setting boundaries for what a YC *should not do*, also meant that the young person’s needs could become fragmented.

We have witnessed young people being referred from BUP, for example, with this way “we can work with your depression here at BUP, but you can go to the youth clinic and talk about your gender identity”. That young people are divided in that way. (Psychologist, YC2)

The above quote illustrates how health system barriers imply that different parts of the person are partitioned, separated and stretched across places and contacts. This is the antithesis of holistic approach whereby the various dimensions of a young person’s health care needs are integrated into the “whole person”.

## Discussion

The role of Sweden’s YCs in mental health is complementary to efforts in other areas of the care sector. Professional staff in YCs work in a holistic manner linking across sexual and reproductive health and mental health. The focus on validating young people’s feelings and experiences and the promotion of good health and positive well-being is paramount. The YC staff try to “normalize” mental health problems and illnesses as far as possible. Young people are seen as active agents and are encouraged to express their own voices and opinions. Parents and guardians are seen as secondary actors.

Staff at the YCs acknowledged that they cannot address the entire range of possible mental health problems and that they must therefore collaborate with other care services. However, there are challenges in forming and maintaining such collaborations, not the least of which include identifying diverse needs and dealing with shortcomings in specialist health care.

### What can other care services that work with young people’s mental health learn from the YCs’ way of working?

Internal and external evaluations and qualitative research conducted in Sweden’s YCs have shown that young people who visit YCs place high value on the care that YCs provide (Föreningen för Sveriges Ungdomsmottagningar, 2015; Sundberg et al., 2021; Thomée et al., 2016; Thomson et al., 2022). They see YCs as inviting and welcoming safe places which can be empowering (Sundberg et al., 2021). Worldwide although health care services strive to be appreciated by young people, there is the perception that this is rarely achieved (Hargreaves et al., 2015; Tylee et al., 2007). The finding in this study that Sweden’s YCs are both well utilized and valued by young people is therefore notable, and in line with findings from studies with young people who perceive youth clinics as “a safe place” (Thomson et al., 2022; Waenerlund et al., 2020).

We argue that the “youth mission” approach could be more widely adopted to make other services more accessible and trusted by young people. Suggested strategies include delivering holistic care, acknowledging young people as active agents, having flexible services, maintaining a low access threshold, and working in multidisciplinary teams. The YCs take opportunities to meet young people and promote their mental health through engendering and fostering safety and trust. International research shows that in many health care meetings young people can feel that they are being judged and consultations are stressful (Gulliver et al., 2010; Plaistow et al., 2014). Positive meetings with care professionals, like the ones described in YCs, can be health-promoting.

Mental health problems that arise during youth and adolescence are costly in the current and longer term—both for the individual and society (WHO, 2021). The broad acceptance of YCs in Sweden reduces perceived stigma associated with seeking mental health support, and helps to build trust in the health care system overall.

In the 2021 report “Mental Health of Adolescents” WHO advised on the need to avoid institutionalization and overmedicalisation, prioritize non-pharmacological approaches and respect the rights of children and youth (WHO, 2021). Key features of Sweden’s YCs, such as the low access threshold, recognizing young people as autonomous active agents and limiting direct involvement with parents, are essential for the promotion of youth friendliness (Thomson et al., 2022).

### Is it possible to treat mental health problems and to promote mental well-being ‘under the same roof’?

The professional staff who were interviewed for this study commented that because of the low access threshold, young people with a wide range of needs attend Sweden’s YCs. When young people present with severe psychiatric problems this is a challenge because such conditions are strictly outside the roles and responsibilities of YCs -with some exceptions whereby some YCs are formally assigned a first line mandate to deal with such. If YCs take on young people with psychiatric diagnosis without the needed resources, other young people risk receiving less care and attention. But if they do not attend the needs of the former, then what happens with the mantra “nothing is too big or too small for YCs”? Taking such a decision (who to accept, who to exclude) can be challenging at the operational level, and contradicts the mission to care for all.

The challenge of providing treatment for psychiatric diagnoses within services that focus on the promotion of mental health is not unique to YCs. Alternative models of “integrated community mental health hubs” for young people are reported in the international literature (Henderson et al., 2023; Malachowski et al., 2019) and they face similar challenges to YCs in Sweden. For example, due to resource constraints, they too struggle to meet the needs of young people with psychiatric diagnoses that exceeds what the care service was mainly designed to handle (Settipani et al., 2019). Models such as “HeadSpace” in Australia have met this challenge by expanding the services they offer at a selected number of dedicated clinics in which there are specialized resources with the capacity to treat different forms of psychoses (Rickwood et al., 2019).

There are strong arguments to be made for resourcing Sweden’s YCs to enable a broader range of mental health care to be offered within a single trusted service. The benefits would include less fragmentation, a more holistic care perspective and easier and more transparent navigation across the health care sector. This would also deliver greater continuity across the mental health spectrum (Baroudi et al., 2020; Keyes, 2005). Young people’s mental health needs could be met within a single service model. This warrants serious attention by policy- and decision-makers.

Implementing the above model would not only require increased resources but a reorganization of the systems currently in place. Research in other countries has shown that there are limits regarding how much extra services can be offered within given resources without lowering the quality of care. As Setippani emphasizes: “the complexity of managing numerous objectives and components within comprehensive integrated community-based youth service hubs for mental health may limit the ability to fully address each aspect simultaneously” (Settipani et al., 2019). Previous research in Swedish settings found that increasing the focus on treatment and specialization can mean that less attention is directed towards mental well-being (Baroudi et al., 2020).

## Methodological strengths and limitations

Given the scope of the topic and the diverse questions it raises, it was appropriate to conduct twenty plus individual interviews with YC professional staff. A strength of the study was the breadth and depth of the information collected from professional staff at the YCs.

Individual members of the research group brought a range of experience in fields covering mental health, public health, psychology, and sexual and reproductive health. These diverse backgrounds fostered creative and open discussion and drew out different perspectives and viewpoints during the analysis stage.

The decision to focus on three YCs may be seen as a limitation. However, the clinics were all different and situated in diverse parts of Sweden, thereby also generating a mix of viewpoints from the staff, as active participants. In addition, even if the focus was on three clinics, the staff in region 1 and 2 rotated through all the clinics in their region (13 counting both regions) and in region 2, staff worked in at least 2 YCs each. This broadens the spectrum on the working places been represented.

This study only analysed the perspectives of professionals. We expect to build upon this research through exploring also the perspective of young people.

## Conclusion

YCs support not only young people’s sexual and reproductive health but also their mental health. They constitute a multiprofessional, safe, and trusted service that meets the needs of young people across many areas of Sweden. The way they approach young people could be an inspiration for other healthcare services in Sweden.

The aim of the YCs is to offer a low threshold easy to access for all young people. However, this means that youth with severe mental health problems also seek help and support at YCs which might not be prepared and sufficient resourced to handle these cases. Facing with a mistrust in the workings of specialized services, YCs are left with the ethical stress of stretching their work responsibilities beyond what they can do or referring young people to an array of unreliable services. This dilemma needs to be further analysed.

YCs can become an important and health promoting part in the youth mental health system if (and only if) other parts of the system is reasonable resourced. If not, the current tension in the system will remain and threaten youth centred services to sink under the workload.

## Data Availability

The dataset analysed during the current study is not publicly available because it contains personal information, but it is available from the corresponding author on reasonable request.
